# 3D vertical nanostructures for enhanced infrared plasmonics

**DOI:** 10.1038/srep16436

**Published:** 2015-11-10

**Authors:** Mario Malerba, Alessandro Alabastri, Ermanno Miele, Pierfrancesco Zilio, Maddalena Patrini, Daniele Bajoni, Gabriele C. Messina, Michele Dipalo, Andrea Toma, Remo Proietti Zaccaria, Francesco De Angelis

**Affiliations:** 1 Istituto Italiano di Tecnologia – Via Morego, 30, I-16163 Genova, Italy; 2 University of Pavia, Physics Department – Via Bassi, 6, I-27100 Pavia, Italy; 3 University of Pavia, Department of Industrial and Information Engineering – Via Ferrata, 1, I-27100 Pavia, Italy

## Abstract

The exploitation of surface plasmon polaritons has been mostly limited to the visible and near infrared range, due to the low frequency limit for coherent plasmon excitation and the reduction of confinement on the metal surface for lower energies. In this work we show that 3D - out of plane - nanostructures can considerably increase the intrinsic quality of the optical output, light confinement and electric field enhancement factors, also in the near and mid-infrared. We suggest that the physical principle relies on the combination of far field and near field interactions between neighboring antennas, promoted by the 3D out-of-plane geometry. We first analyze the changes in the optical behavior, which occur when passing from a single on-plane nanostructure to a 3D out-of-plane configuration. Then we show that by arranging the nanostructures in periodic arrays, 3D architectures can provide, in the mid-IR, a much stronger plasmonic response, compared to that achievable with the use of 2D configurations, leading to higher energy harvesting properties and improved Q-factors, with bright perspective up to the terahertz range.

Nowadays, the application of plasmonics spans over many different fields such as sensing through enhanced spectroscopy^1^,^2^,^3^,^4^,^5^,^6^, metamaterials^7^,^8^,^9^,^10^, fine engineering and tuning of optical properties^11^,^12^, chemical catalysis^13^,^14^,^15^, lasing^16^,^17^, AFM and scanning probe spectroscopies^18^,^19^,^20^, quantum plasmonics^21^,^22^ and many others^23^,^24^,^25^,^26^,^27^. Looking beyond the details of specific applications, one can see that in most cases the success of plasmonic nanostructures relies on their capability of effectively (i) harvesting the electromagnetic radiation, and (ii) confining it in so called *“hot spots”* of subwavelength size, usually few nm, where (iii) the electric component of the field is strongly enhanced. So far however, despite the fascinating achieved results, the exploitation of plasmons has been mainly limited to the visible and near infrared (IR) ranges of the electromagnetic spectrum, because the optical properties of metals intrinsically lead to a reduction of the plasmonic spatial confinement in the IR region. Moreover, in the low frequency (mid-IR) limit the plasmonic relaxation time τ_pl_ becomes comparable to the optical cycle time τ_opt_cycle_, thus approaching the limit for coherent plasmon excitation. A good indication to describe this loss in coherence may therefore be found in the quantity Q = ω/Γ, where ω is the energy of the incoming photon and Γ is the damping constant. Currently, on one side, the search of novel materials – alternative to noble metals and offering improved performance for IR plasmonics, for instance at the telecom frequencies – is a hot topic in nowaday’s literature^28^,^29^,^30^,^31^; on the other side, classical optical approaches such as diffractive coupling or optical gratings^32^,^33^,^34^,^35^,^36^ have been exploited to improve the plasmonic response, independently from the material of choice. In fact, lateral scattering can be used to create constructive interference between the incoming light and the nanostructured surface, which in this case may work as an optical grating: when nanostructures are arranged in an ordered array, the local electric field acting on each nanostructure sums-up with the incoming external field and with the retarded dipoles produced from surrounding nanostructures (i.e. the scattered fields). If the distance among the nanostructures and the angle of incidence of light satisfy proper geometrical conditions^32^,^37^, the retarded dipoles add in-phase, giving rise to a grating effect^38^,^39^,^40^,^41^,^42^. This constructive interference is known to produce collective plasmon excitations with narrower resonances and higher local electric fields, thus partially overcoming the mentioned limitations of plasmonics in the IR regions.

In this work we show that passing from conventional 2D to 3D architectures enables stronger plasmonic response, longer lifetime, higher harvesting capabilities, and higher field enhancements in the IR region. Both with numerical calculations and with experimental characterizations, we suggest that the physical principle relies on the combination of far field and near field interactions between neighbouring antennas, and that this is abet by the out-of-plane geometry. To better explain these concepts, we first analyse the changes in the optical behaviour that occur when passing from a single on-plane nanostructure to a 3D out-of-plane nanostructure. Then we show that by arranging the nanostructures in periodic arrays (grating effect), 3D architectures intrinsically promote a much stronger plasmonic response, compared to that achievable with the use of 2D configurations, even in the mid-IR.

## Results and Discussion

**Figure 1:**
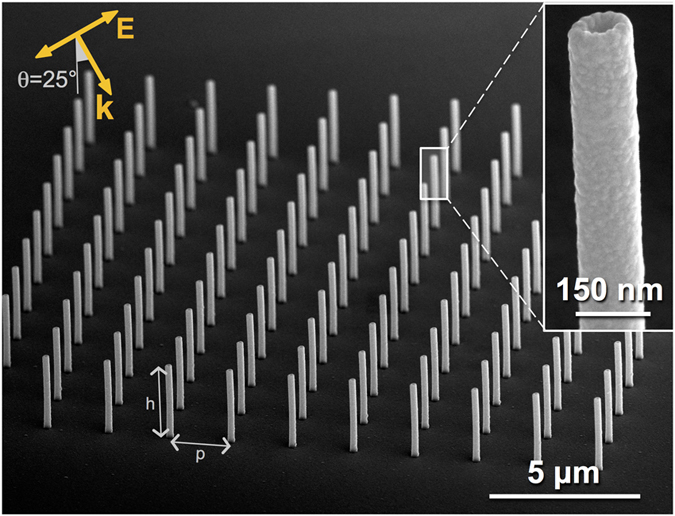
SEM image of an ordered array of silver vertical nanotubes, with a given length (height *h* varied between 0.85 μm and 7.5 μm) and inter-antenna distance (pitch *p* varied between 2 μm and 9.5 μm). IR broadband illumination (impinging angle θ = 25°, TM-polarization) triggers LSPRs on the longitudinal axis; inset: magnified view of a 3D hollow nanocylinder. Inner cavity and outer diameter (80 nm and 140 nm respectively) are kept constant in all specimen.

The proposed device consists of noble metal out-of-plane nanotubes, whose main axis comes out from the substrate (see Fig. 1). The fabrication method of 3D vertical nanoantennas is based on the combination of focused ion beam milling and secondary electron lithography and has already been described elsewhere^43^. For completeness, a detailed description of the fabrication protocol is also reported in the methods section. The result is high-aspect-ratio, hollow, vertical polymeric scaffolds extruding from the plane and positioned with nanometric precision, which are then coated with a 30 nm silver layer to trigger plasmonic response, as shown in Fig. 1.

We may notice some peculiarities enabled by the novel manufacturing approach: in particular, structures have a toroidal instead of a squared-like cross section, they are hollow instead of bulk, vertical (out of plane) instead of planar, short-circuited by metal instead of isolated, and they are surrounded by a homogeneous environment (no substrate influence, which is known to damp the plasmon oscillation^44^,^45^).

**Figure 2:**
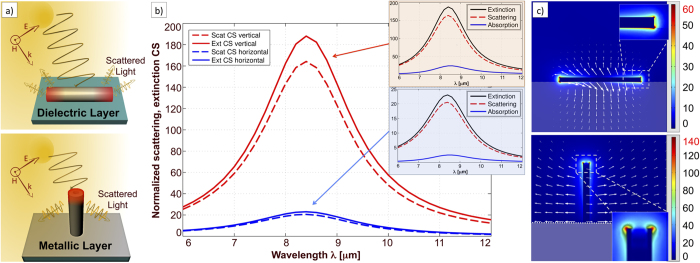
(**a**) Sketch comparing a planar nanorod with an out of plane single hollow nanocylinder: the different spatial arrangement and the presence of the metallic substrate are responsible for an efficient harvesting and a convenient on-plane scattering; (**b**) extinction (E) and scattering (S) magnitude from a vertical (V) and a planar (P) nanoantenna (normalized cross sections calculated as the ratio between scattered power and power impinging on the cross sectional area of the structures); (**c**) numerical calculations (see text and SI#3) evidence a monopolar electric field distribution (colour plot, intensity normalized to incident field) and a convenient radial energy flow (arrows represent the Poynting vector) from vertical nanotubes (bottom figure), whereas planar configurations (top figure) exhibit a standard dipole mode and a power flow mainly directed into the substrate. In both layouts, the impinging wavelength is λ_res_ = 8.5μm.

In order to compare the optical response of a single planar antenna with a 3D-vertical single architecture, we performed numerical calculations (COMSOL Multiphysics software, details in Supporting Information SI#1) of the two systems. Figure 2a sketches the two configurations, while Fig. 2b,c respectively illustrate the far field and near field responses. In particular, Fig. 2b shows the simulated extinction and scattering coefficients of a single vertical nanotube (silver, H × 2R = 1700 × 160 nm, silver underlying substrate, angle of incidence ϑ_inc_ = 45°), compared to a single planar nanorod with the same volume (silver, L × 2R = 2400 × 134 nm, angle of incidence ϑ_inc_ = 45°, silicon nitride underlying substrate), both resonating at approximately λ = 8.5 μm. Despite the apparent flaw of triggering the plasmonic response in planar structures with a non-vertical wave vector, the choice of tilting the source for both arrangements allows a fair comparison of scattering and extinction cross-sections. Also the choice of considering a planar nanocylinder, is ruled at this stage by the need of providing a direct comparison exclusively of the two structures’ arrangement in space. This is formally true because, at the studied energies, hollow structures can be considered in good approximation as bulk monolithic metallic cylinders (see SI#2 for calculations). At last, the simulation domain was kept to 2 times the maximum wavelength, and we verified that no change in the results was obtained by further increasing the simulation region.

As it can be seen, the extinction efficiency in vertical nanotubes is more than eight times higher compared to planar nanostructures (Fig. 2b), while the electric field enhancement increases from 60 to 140 in amplitude (Fig. 2c). Furthermore, by investigating the two components constituting extinction (absorption and scattering), we notice that such a strong increase is due to the scattering term rather than from absorption. A very important role seems to be played by the metallic substrate for the case of the out-of-plane antenna. In fact, while the electric field norm profile for the planar antenna (Fig. 2c top) represents a standard dipole configuration, when the out-of-plane configuration is considered (Fig. 2c bottom) a monopole-like modal response is observed. Such a monopole-like mode exhibits a charge density at the tip-end which is doubled with respect to that of a standard dipole, thus increasing the electric field enhancement of a factor two (see SI#3 for details). This feature, together with the refractive index homogeneity of the surrounding medium (the vertical antenna is in air, no plasmon damping due to the substrate), makes the vertical antennas capable of higher field enhancement. As mentioned above, it passes from 60 to 140 in amplitude.

Still analysing Fig. 2c, we observe that when the incoming wavelength matches the resonance of the system, the vertical architecture in combination with the metal layer at the base induces a rotation of the incoming field, making the Poynting vector streamlines of the scattered field parallel to the substrate surface. Therefore, opposite to planar arrangements, transmission and back reflection of the incoming light are negligible. To summarize the analysis of the isolated nanostructure, we underline that the theoretical results shown in Fig. 2 suggest an intrinsic improved performance of 3D nanoantennas, compared to planar architectures. Furthermore, when nanostructures are arranged in periodic arrays to improve the plasma-like response, the intrinsic features of the 3D configuration can synergistically combine with an engineered spatial layout, leading to additional advantages and further improved optical outputs. In fact, in a periodic array of 2D planar antennas, incoming light is mainly back reflected or transmitted. On the contrary, in 3D configurations, thanks to the enhanced lateral scattering, light is continuously re-scattered along the substrate plane and power flow is redirected within the volume occupied by neighbouring nanostructures, thus promoting a more effective harvesting of energy, and thus an improvement in field enhancement. This is discussed in the following paragraphs, where we first analyse optical properties of 3D periodic arrays and then conclude with a quantification of resonance quality factors.

**Figure 3:**
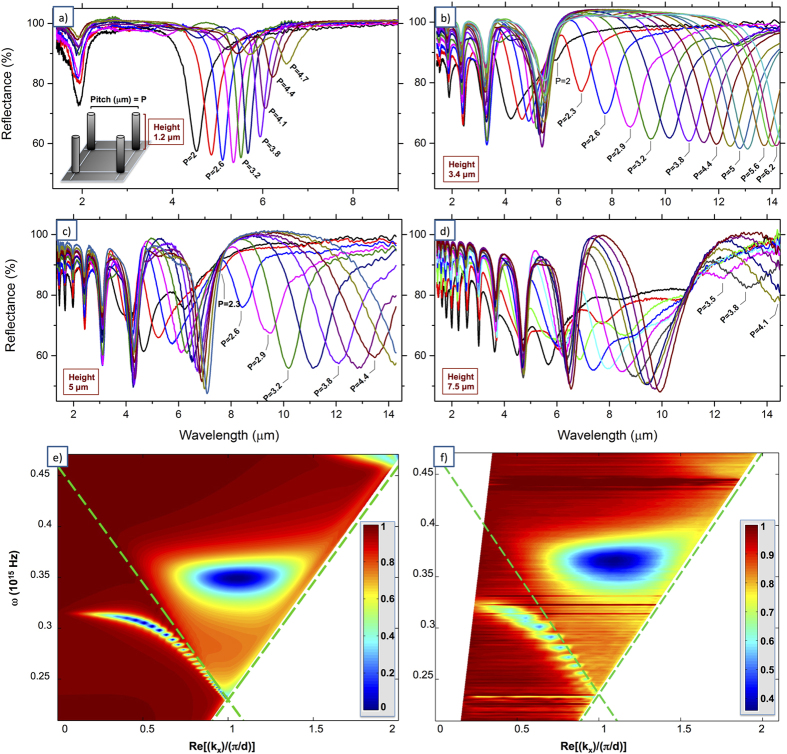
(**a**–**d**) FTIR spectroscopy characterization of ordered arrays of vertical hollow nanotubes, for different heights (h = 1.2–3.4–5.0–7.5 μm) and different interantenna distances (p). Among the most evident traits of 3D arrays of nanoantennas is the extraordinarily high number of resonance modes – up to 12 in the studied IR range, for 7.5 μm tall structures (Fig. 3d) – and an almost complete energy absorption (50% of unpolarized light) even for relatively small active areas (5 × 5 structures, see text for details); (**e**,**f**) numeric calculations and experimental data for angle-resolved specular reflectance (R0) spectra, nanoantenna array height h = 1.2 μm, pitch p = 4.1 μm (simulations: impinging angles between 0°–80°, experimental: 10°–70°).

With this aim, we fabricated ordered arrays of silver nanotubes resonating from 4 to 14 microns. Antenna heights are varied in the ranges *h* = (0.85–7.5) μm and inter-antenna distance (pitch, *p)* in the range *p* = (2–9.5) μm. Other parameters are kept constant: spatial square lattice arrangement, number of structures (5 × 5), inner diameter d_1_ = 80 nm, outer diameter d_2_ = 140 nm, silver layer thickness t = 30 nm. Experimental characterizations were carried out with a commercial micro-FTIR setup (ThermoFisher iS50) equipped with a cassegrain condenser lens which mimics a 15×, NA = 0.58 objective, in the spectral range between 1.4 μm and 15 μm. A background spectrum on a nearby unpatterned silver area is acquired before each measurement, while the fixed number of elements in every matrix allows us to compare efficiency and optical response from different arrays without corrections. Figure 3a–d shows the experimental spectra of **3**D hollow nanotube arrays at four representative different heights (*h* = 1.2 μm, *h* = 3.4 μm, *h* = 5 μm and *h* = 7.5 μm).

First we notice that for longer antennas a large number of dips can be observed in the reflection spectrum. Numerical calculations show that they are associated with longitudinal resonances (Fabry-Perot-like modes) of different orders. For instance, structures as high as 7.5 μm host up to 12 orders of resonance in the studied energy range (Fig. 3d). We also point out that measurements were carried out with unpolarized light, whereas vertical nanotubes can couple mainly photons having a component of the electric field parallel to the nanotube axis (usually referred to as *p*-polarization or TM-polarization). This means that a dip of 50% in the reflection spectrum corresponds to an almost complete harvesting of the *p-*polarization (see SI#4). It is also important to mention that the data we present here is collected from matrices of no more than 5 × 5 elements, and the needed acquisition time can be as low as ten seconds, demonstrating that the vertical elements can produce a very strong optical output also when fabricated in a very small active area. The most immediate application concerns IR surface-enhanced biosensing, where a desired feature is to have strong signal from small active areas^42^,^43^,^45^.

**Figure 4:**
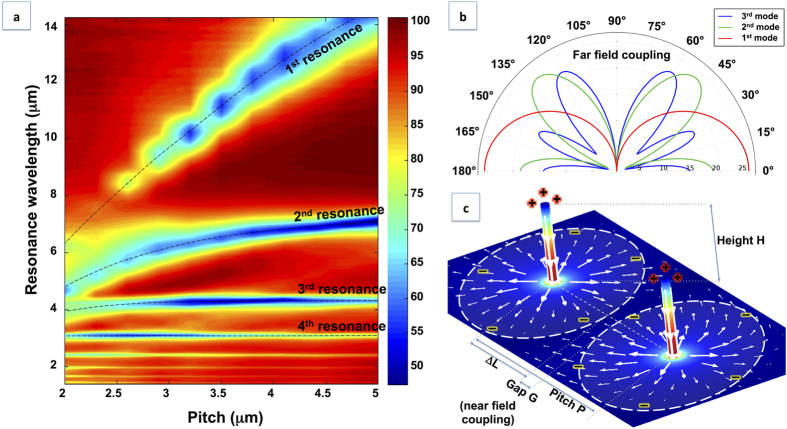
(**a**) color plot summarizing reflectance as a function of wavelength and interantenna distance, for arrays of 5 μm long nanoantennas (dataset from Fig. 3c). Lower order modes (1st and 2nd) exhibit a strong dependence of resonance wavelength with pitch, due to a convenient energy flow parallel to the substrate (**b**) and a near field coupling into the substrate (**c**); In detail, (**b**) shows far field coupling, mediated by scattered EM fields, for the first three order modes (1^st^ monopolar mode parallel to substrate, 2^nd^ dipolar mode mostly directed outwards with an angle of 50°, 3^rd^ order mode mostly with an angle >60°), while (**c**) shows near field coupling, mediated by charge density oscillation (white arrows) into the metallic substrate (simulation: COMSOL multiphysics). As the pitch is decreased and structures are placed in a tight spatial arrangement, repulsive forces of in-phase oscillation contrast external excitation, damping the optical resonse (see 1st order resonance in (**a**), pitch < 3 μm).

By looking more in detail the data reported in Fig. 3, we notice that the position of some of the resonances shows a remarkable dependence on the pitch. In particular, when the pitch is smaller than the wavelength of resonance, we observed a very strong blue shift for decreasing pitch values. For the layouts and the range of wavelengths we investigated, it mainly occurs for the first and second order of resonance. This behaviour is summarized in Fig. 4a, where the magnitude in reflectance from all spectra in Fig. 3c is reported as a function of wavelength and pitch.

For completeness, we also measured the optical response of a single arrangement (height h = 1.2 μm and pitch 4.1 μm) with the change of the impinging angle. To do so we fabricated structures over an area as large as the illumination spot size of the instrument (500  ×  500 μm^2^) and used a custom homemade micro-reflectometer setup coupled to a FTIR spectrometer (Bruker IFS66). Figure 3d,e show numerical calculations and experimental data of reflectance spectra, for wave vectors entering between 0° and 80° (see also SI#4 for experimental unprocessed data). One dip is clearly dispersive while increasing the angle, as a monotonous function, red-shifting in wavelength and asymptotically approaching the first Wood-Rayleigh anomaly along the x direction; the other dip shows no change in λ_res_ but increases in magnitude, showing a local maximum for an angle of 45°.

Coming back to the dataset in Fig. 3(a–d), the strong dependence on the pitch is not surprising. In fact, as introduced above, in a periodic arrangement the local electric field acting on each nanostructure sums-up from the incoming external field and from the retarded dipoles produced from surrounding nanostructures (i.e. the scattered fields)^32^. Therefore, each spatial arrangement can promote a constructive interference only for a given wavelength. As expected, for smaller values of pitch, the dips in the reflection spectra shift to shorter wavelengths. This behaviour is usually observed in periodic arrangements of nanostructures aimed at tuning and improving the plasmonic response^38^,^42^,^46^. However, in this case such a collective coupling is further strengthened by the strong lateral scattering provided by the 3D arrangement, which redirects the Poynting vector streamlines of the scattered field parallel to the substrate surface and onto neighbouring structures, and by the presence of the conductive layer on the substrate. Figure 4b shows the polar diagram of a vertical nanoantenna, suggesting how in-plane scattering is more effective for low-order resonance modes: this accounts for the optical behaviour of ordered arrays (see datasets in Fig. 3c,d).

Furthermore, as in the case of isolated nanostructures, we analysed the role of the metal layer short-circuiting the vertical antennas by numerical calculations. Figure 4c reports the charge density distribution on the conductive substrate plane for an array of antennas, extracted from the simulation (height *h* = 1.2 μm, pitch *p* = 2.6 μm, first order of resonance at λ = 5 μm).

As it can be seen, the monopole’s oscillating charge, mirrored into the substrate, becomes subject to the repulsion of in-phase nearby oscillating electron waves, coming from neighbour antennas. When the distance between the antennas is comparable with λ/2 this repulsive force produces a strong interaction regime that is responsible for the rapid blue-shift of the resonance. In other words, the metallic layer enables a near field coupling for antennas, which are now virtually separated from a nanometric gap in a planar dimer-like configuration. This explains also the fact that very densely packed layouts exhibit a reduced intensity in absorption (see, for instance, spectra of pitches 2.3–2.9 in Fig. 3c) that can reach a suppression of lowest-order modes for very short pitches, when the repulsion of electron waves in the substrate exceeds the external loading excitation force.

**Figure 5:**
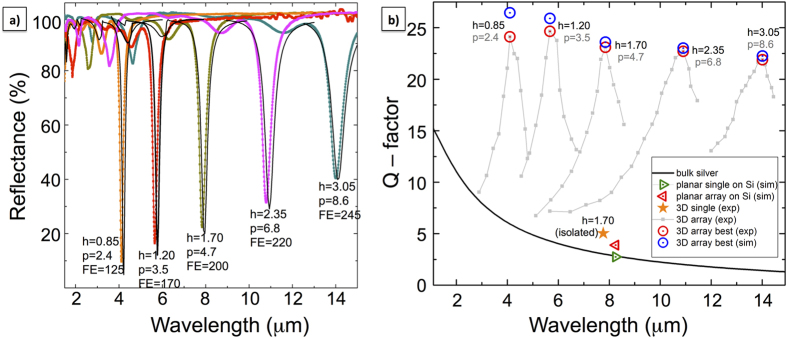
(**a**) Best experimental FTIR reflectance plots (coloured/dotted) from arrays of silver nanotubes at different heights h: 0.85 μm, 1.20 μm, 1.70 μm, 2.35 μm, 3.05 μm. The corresponding numerical results are shown with black/solid curves. Field enhancement amplitudes (FE), extracted from simulations, are reported close to the minima. (**b**) Quality factors calculated from experimental curves of panel (a) as Q = λres/FWHM, together with the ones computed for 3D isolated antenna, 2D isolated antenna, a 2D array in its most performing layout (simulation geometries extracted from ref. 32) and the bulk value.

Driven by the previous arguments, we conclude now with a quantification of the improved performances in ordered arrays of nanoantennas, with respect to optimized planar devices and single vertical structures, as reported in Fig. 5. As it is well-known, the quality of plasmonic response can be evaluated by the ratio between real and imaginary part of permittivity, *Q* = *−*ε_real_/ε_imaginary_^44^. In the IR range it can be approximated as *Q* = ω/Γ, where ω is the energy of the incoming photon and Γ is the damping constant, mainly given by bulk scattering mechanisms. For each antenna height, the best optical response (yielding highest quality factors Q, spectra extracted from the dataset in Fig. 3) is shown in Fig. 5a.

Numerical computations carried out with COMSOL Multiphysics are also reported (black solid lines), showing very good agreement with experiments, and remarkably high electric field enhancements, progressively growing in the IR region with a best value of 245 (in amplitude) at around 14 microns. The corresponding values of the best-measured quality factors are reported in Fig. 5b (red bull’s eye), highlighted over the values exhibited by non-optimized gratings (light gray background curves). Very good experimental quality-factors exceeding values of 20–25 in the whole mid-IR range are clearly evidenced, with an almost constant trend that opens good perspectives in the far-IR and terahertz region. As a direct comparison, Fig. 5b also shows the quality factor of a single, vertical antenna (experimental measurement), and the numerically calculated values for a single planar nanoantenna and an optimized array of nanontennas on a silicon substrate. As reference, the quality factor of surface plasmons in bulk silver is plotted (dark black line).

## Conclusions

To summarize, we demonstrated that by realizing 3D nanostructured materials with out of plane architectures and high aspect ratio, it is possible to strongly improve the plasmonic response in the IR range. An isolated vertical nanoantenna exhibits an extinction efficiency increased of a factor 8 with respect to the planar one, thanks to a strong lateral scattering and monopole-like charge density distribution induced by the metal layer at the base of the antenna. In periodic arrays, the strong lateral scattering, which redirects the light towards neighbour antennas, promotes a far field coupling which is intrinsically more effective than that achievable on planar layout. In addition, the metal layer short-circuiting the antennas enables a near-field coupling even for antennas that are virtually separated from a micrometric gap, boosting optical performances. The combination of both effects produces narrow resonances (quality factor as high as 20–25 in the whole mid-IR range) and high local electric fields even in small periodic arrays of few elements (5 × 5). Moreover, the very small surface area and the very short integration time required to obtain a good optical response make the presented device suitable for practical applications, such as efficient enhanced IR spectroscopy. At last, these architectures show the harvesting and confining efficiency of electromagnetic radiation typical of plasmonic systems operating in the visible range, thus suggesting brighter perspectives for IR plasmonics.

## Methods

The method followed to fabricate samples is introduced and fully described in ref. [43].

Among its features is a fine control over shape and size, yielding uniform, high aspect-ratio and mechanically resistant hollow nanotubes.

The principle relies on FIB-generated secondary-electron lithography in optical resists.

In our case, s1813 optical resist is spun on 100 nm thick Si_3_N_4_ membranes and soft-baked at 100 °C for 5 minutes. In the range spanning between 1.5 μm and 7.5 μm, thickness is controlled by tuning spinning time and velocity; below 1.5 μm resist must be diluted with anisole. A good control over polymer deposition is crucial, because it fixes the height of the nanostructures at the end of fabrication. Membranes are then patterned from the backside using a Focused Ion Beam (Helios Nanolab600, FEI company), operated at 30 keV (current aperture: 40pA, dwell time: 500 μs, number of repetitions: 850 passes). Due to the high dose of low-energy secondary electrons induced by ion beam/sample interaction, a 40 nm thick layer of resist, surrounding the milled hole, becomes highly cross-linked and insoluble to most solvents. After patterning, the sample is developed in acetone, rinsed in isopropanol and dried under gentle N_2_ flow. A mild oxygen plasma (process time: 2 minutes, RF power: 100 Watt, gas flow: 25 sccm) thins the polymeric skin down to 10 nm.

A 30 nm thick layer of silver is deposited by sputtering the sample, tilted 60° with respect to the vertical and rotated, guaranteeing an isotropic coating on both the sidewalls and the base. At last, the metal layer is annealed in inert (N_2_) atmosphere for one hour at 180°C, coalescing the polycrystalline silver nanoparticles into larger uniform domains.

## Additional Information

**How to cite this article**: Malerba, M. *et al.* 3D vertical nanostructures for enhanced infrared plasmonics. *Sci. Rep.* **5**, 16436; doi: 10.1038/srep16436 (2015).

## Supplementary Material

Supplementary Information
