# Supplementary material for: 3D vertical nanostructures for enhanced infrared plasmonics
Source: Sci Rep. 2015 Nov 10;5:16436. doi: 10.1038/srep16436 (PMC4639734; doi:10.1038/srep16436)
Supplement: Supplementary Information [file srep16436-s1.doc]

Supporting Information

Title: 3D vertical nanostructures for enhanced infrared plasmonics

Mario Malerba ^‡^, Alessandro Alabastri ^‡^, Ermanno Miele ^‡^, Pierfrancesco Zilio, Maddalena Patrini, Daniele Bajoni, Gabriele Messina, Michele Dipalo, Andrea Toma, Remo Proietti Zaccaria, and Francesco De Angelis^*^

**SI#1 - Calculation Details**

Simulations have been carried out using commercial FEM software (COMSOL Multiphysics). The incident field has been approximated by a tilted TM polarized plane wave. For single antennas calculations Perfectly Matched Layers (PMLs) have been employed in order to truncate the simulation domain. Regarding antenna arrays, lateral PMLs have been substituted by periodic boundary conditions to keep into account both phase shift of the incident field and the interaction with other antennas.

As far as single antennas extinction efficiency is concerned, it has been calculated as the sum of absorption and scattering efficiency. The former derives from the integration of the ohmic losses over the metallic domains of the antenna; the latter comes from the integration of the Poynting vector related to the scattered field over a closed surface surrounding the antenna.

**SI#2. Comparison of hollow and bulk optical behavior in the mid_IR**

Hollow nanostructures can be treated as bulk, solid metallic nanocylinders, due to the negligible dimensions of the cavity, compared to the exciting wavelength. As shown in a previous paper (see calculations presented in figure 5, in *De Angelis et al., Nano Lett., 2013, 13 (8), pp 3553–3558*), optical excitation on the inner polymer-metal interface can be achieved, in a hole of less then 100nm, only with an impinging wavelength λ < 1 μm. Here we point-out that also scattering and extinction cross sections are almost the same in both cases. Figure SI2.1 shows the normalized cross sections for an isolated antenna, baring the same geometries described in the main text, with and without the inner metal cavity.


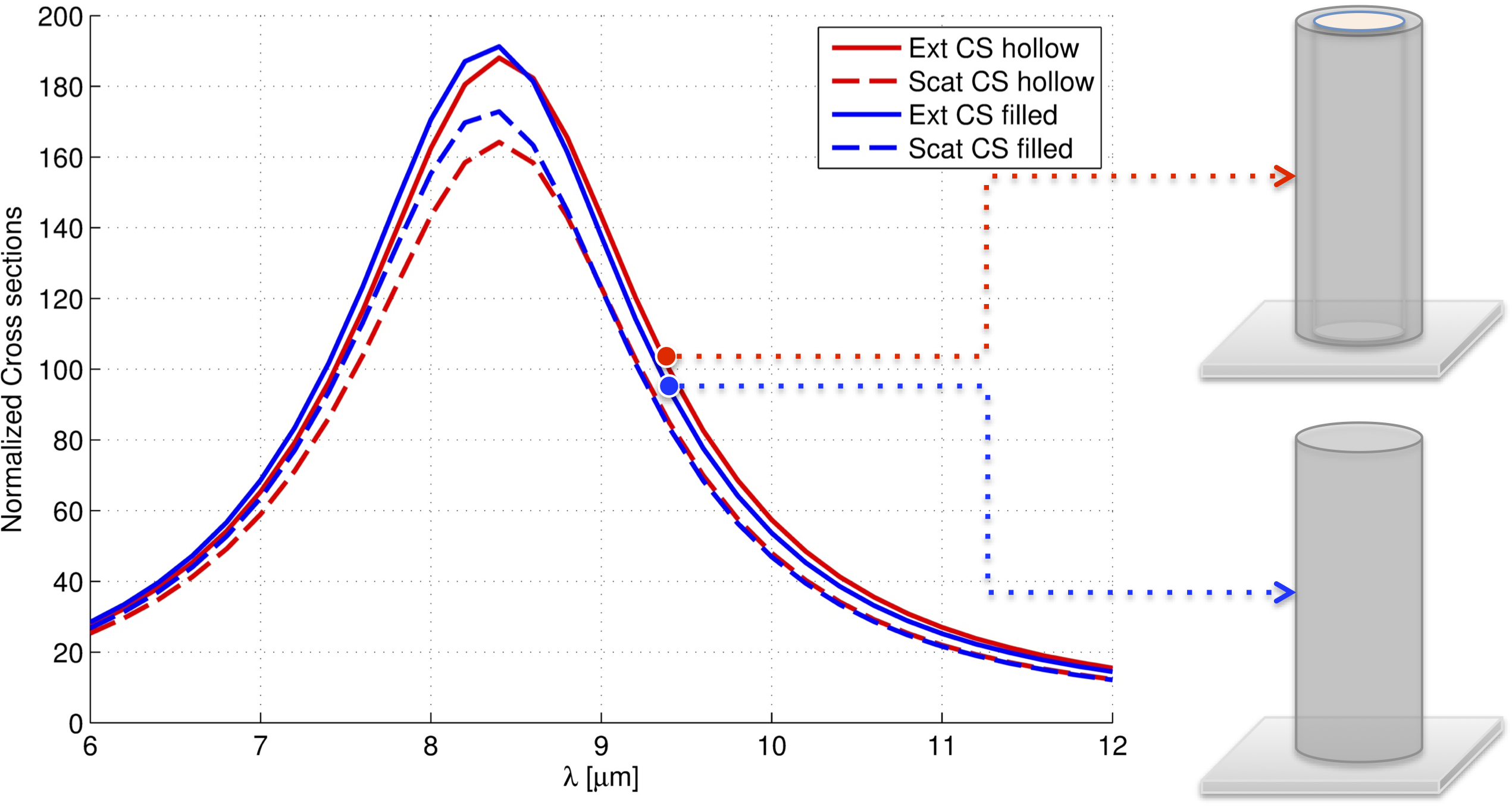


*Figure SI3.1: Extinction and scattering cross sections of bulk (filled) and hollow vertical nanoantennas, normalized to the ingoing wave vector.*

**SI#3. Contributions of an out-of-plane configuration on a metallic layer**

Planar antennas have been deeply studied in the last few years in order to investigate their plasmonic properties. Fabrication techniques for this kind of configuration are well established and thus a lot of work has been done on 2D systems both from an experimental and theoretical point of view. Unfortunately planar antennas intrinsically require a substrate to lie upon and this constrain has been proven to hinder plasmonic properties red-shifting and damping their resonances while increasing the line-widths. This is due to the refractive index mismatch at the interface, which allows the scattered electric field by the antenna to spread into the substrate. This issue can be overcome by fabricating out of plane antennas. In this case the contact area between the structure and the substrate is reduced by a factor proportional to the aspect ratio of the antenna. Moreover the small interaction area with the substrate can be further exploited. In fact, introducing a metallic layer between the antenna and the dielectric substrate allows us to virtually double the length of the antenna; the concept can be understood reminding the concept of image charges (**Figure SI3.1**).


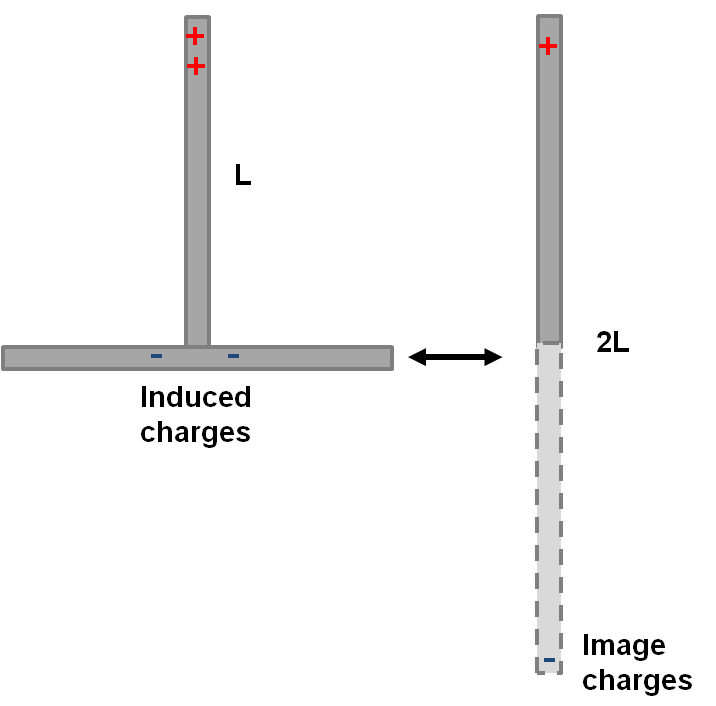


*Figure SI3.2: effect on induced charges when the antenna is placed over a metallic substrate*

When charges accumulate at the tip of the L antenna, charges of the opposite sign are induced in the metallic substrate, which acts as a reservoir. The phenomenon can be read in terms of image charges. Therefore the situation is equivalent to a 2L antenna with no substrate inserted in a homogenous background. Since the electric potential must coincide in both cases and the incident electric field is the same, it follows that charge density at the tip of the L antenna must be twice the density of an independent antenna with a doubled length.


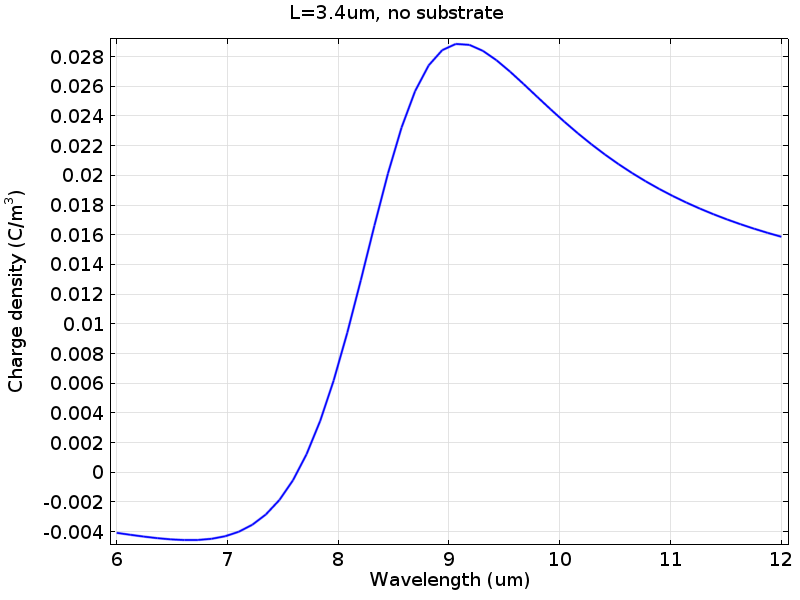

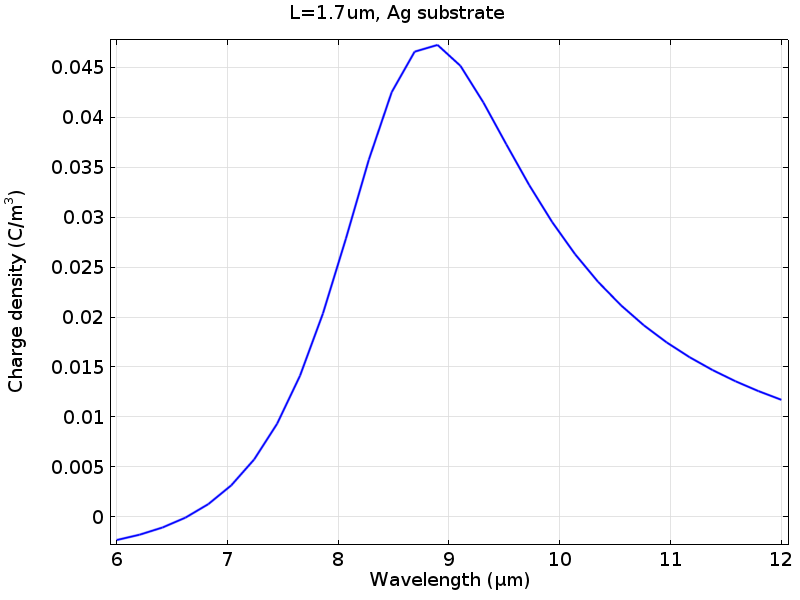


*Figure SI3.3: density of charges accumulated 1nm below the tip end in two cases: (LEFT) antenna with L=3.4um embedded in air and (RIGHT) antenna with L=1.7um placed on a metallic layer.*

In **Figure SI3.2** it is reported the spectrum of the density of charge at 1nm below the end of the tip in two cases. The incident field is a tilted (θ=45°) TM polarized plane wave. At the left we see the plot for a L=3.4um antenna in air, at the right the same plot for a L=1.7um antenna placed over a d=30nm Ag layer. As predicted the resonant wavelength is very close and the charge density ratio is almost 1:2.

Since electric field is generated by accumulated charges, in the second configuration we can obtain a doubled field enhancement at a resonant wavelength which normally would require an antenna twice the height (see **Figure SI3.3**).


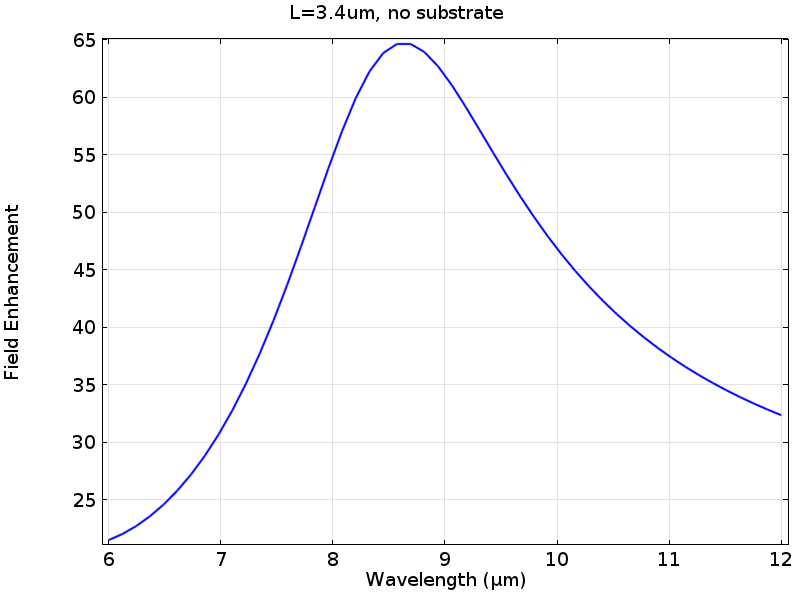

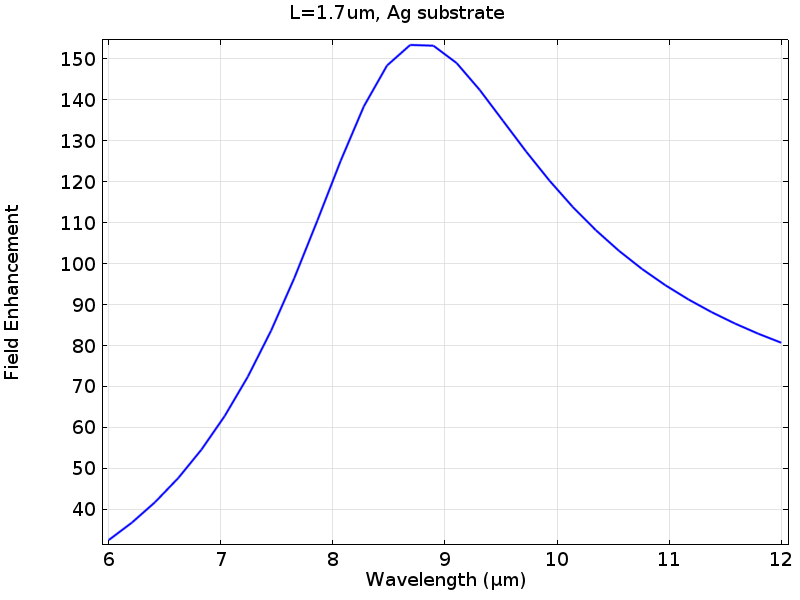


*Figure SI3.4: field enhancement at 1nm above the tip end in two cases: (LEFT) antenna with L=3.4um embedded in air and (RIGHT) antenna with L=1.7um placed on a metallic layer.*

**SI#4. Angle-resolved reflectance spectra from large nanoantenna arrays, and measurements with polarized light.**

Angle-resolved reflectance measurements were performed in the medium infrared range using a homemade microreflectometer setup coupled to a Fourier-Transform spectrometer (Bruker IFS66, with a LNT cooled MCT detector). Typical measurements required 1024 scans with spectral resolution of 4 cm^−1^ and angular aperture of about 2°.

The customized optical bench allows *p*-polarized light to be selected and controlled on the impinging angle, to the detriment of a small illumination spot size (diameter > 300 μm). Figure SI4.1 shows a portion of the 500x500 μm^2^ nanotube array and the raw reflectance data acquired (compare with figure 3f in the manuscript). In particular, the angular dispersion of the Fabry-Perot-like monopolar resonance mode asymptotically approaches the 1^st^ Wood-Rayleigh anomaly along the main lattice axis; the angle-independent peak at 1875 cm^-1^ (λ = 5.33 μm), also found at the same wavelength in figure 3a for the same layout (pitch p = 4.1 μm), corresponds to a 2D grating peak, connected to the triggering of a surface plasmon on the metal baseplate.


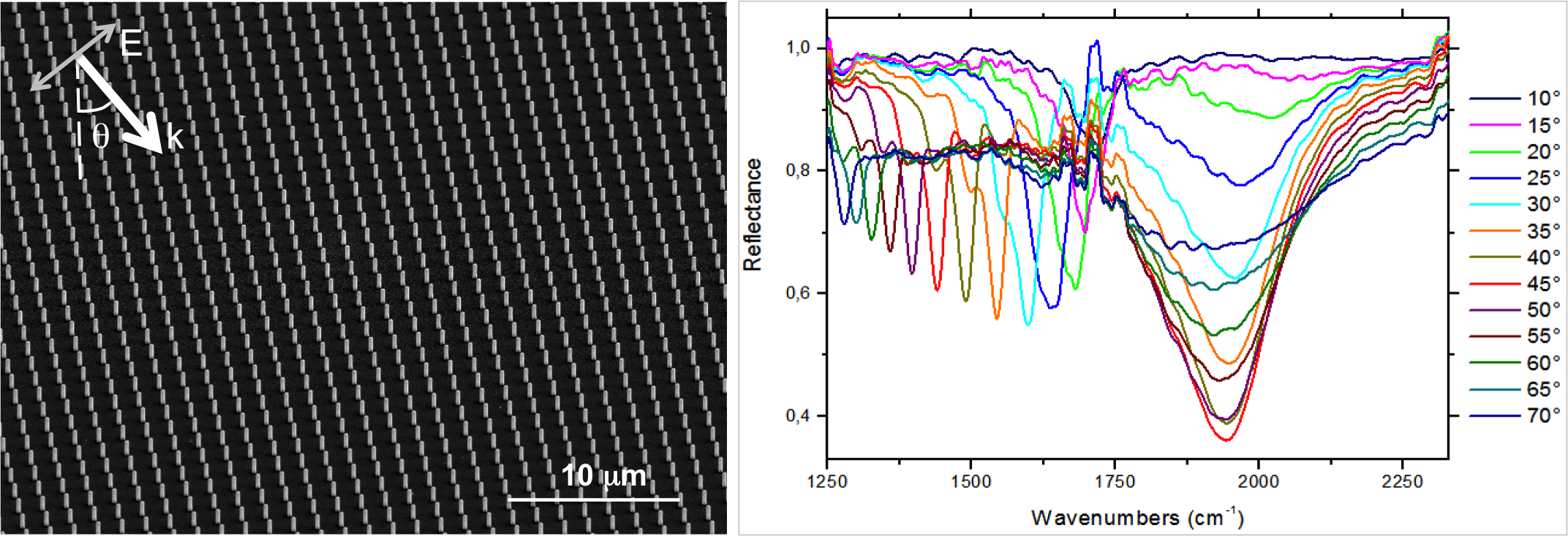


*Figure SI4.1: (left) portion of a large (500x500) μm^2^ and scheme of illumination, using a custom microreflectometer setup (see text); (right) reflectance spectra (raw / unprocessed experimental data), used to produce the color map in figure 3e.*

At last, we wish to validate with experimental proof the statement by which "a dip of 50% in the reflection spectrum corresponds to an almost complete harvesting of the *p-* (or TM) polarization". The same mentioned setup was used for this purpose. An arrangement yielding a broader dip in reflectance compared to the latter – thus less sensitive to the non-negligible angular aperture of the imperfectly collimated beam (approximately 2°) – was fabricated (height h = 1.6 μm, pitch p = 4.1 μm) and measured both with our iS50 micro-FTIR and with the custom microreflectometer setup, using in this case *p*-polarized light impinging with an angle θ=25°. Figure SI4.2 shows the comparison between the two optical outputs: a conveniently polarized beam can be almost completely harvested by the vertical nanoantenna array, with a reflectance dip verging on 10%.


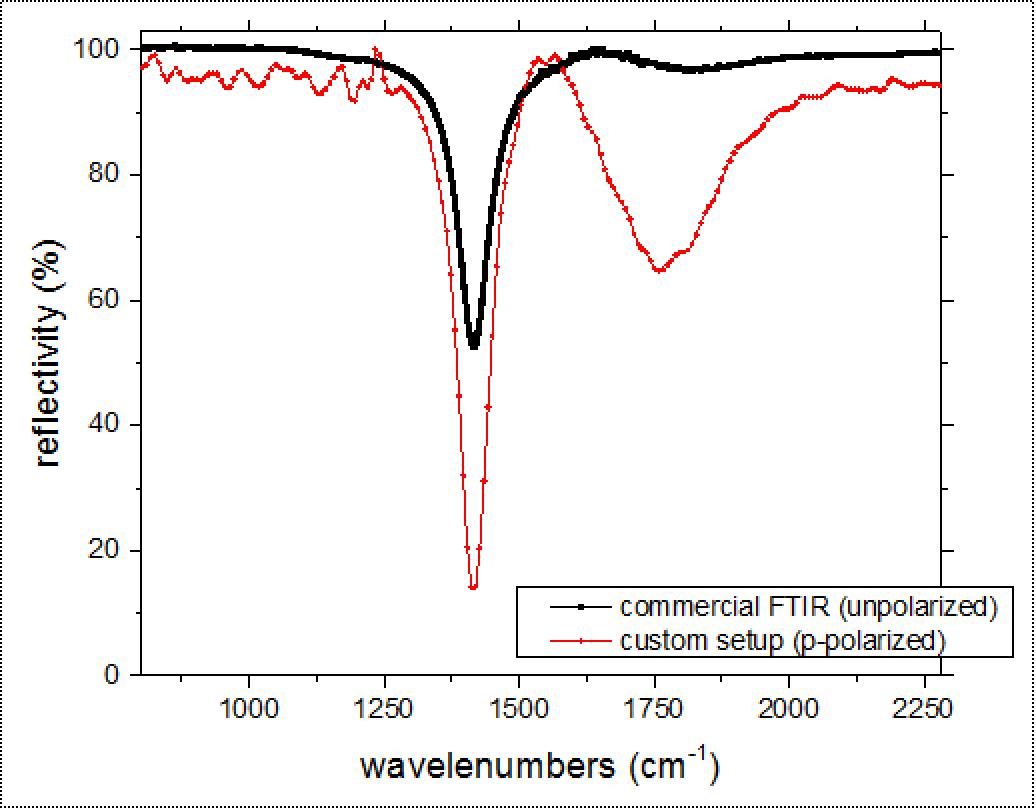


*Figure SI4.2: Comparison of optical output using unpolarized / uncollimated (commercial setup) vs. TE polarized / collimated light. An almost complete harvesting of* p*-polarized light is evidenced in the second case.*
